# Zumba-induced Takotsubo cardiomyopathy: a case report

**DOI:** 10.1186/s13256-018-1696-x

**Published:** 2018-06-10

**Authors:** Sana Chams, Skye El Sayegh, Mulham Hamdon, Sarwan Kumar, Zain Kulairi

**Affiliations:** Department of Internal Medicine, Wayne State University School of Medicine, Rochester Hills, MI USA

**Keywords:** Takotsubo cardiomyopathy, Spironolactone, Anxiety, Catecholamine

## Abstract

**Background:**

Takotsubo cardiomyopathy or stress cardiomyopathy is characterized by transient left ventricular apical ballooning in the absence of coronary occlusion. The underlying pathophysiological mechanism is still unclear but possible causes have been proposed mainly catecholamine cardiotoxicity, followed by metabolic disturbance, coronary microvascular impairment, and multivessel epicardial coronary artery vasospasm. Takotsubo cardiomyopathy accounts for 1–2% of patients presenting with acute coronary syndrome with the majority of patients diagnosed with Takotsubo cardiomyopathy being women > 55 years of age. Here, we discuss the case of a 38-year-old woman presenting with typical chest pain, electrocardiography changes and cardiac markers consistent with acute coronary syndrome, who was subsequently diagnosed with Takotsubo cardiomyopathy.

**Case presentation:**

A 38-year-old healthy American woman with negative past medical history presented to our Emergency Department with chest pain developing while participating in intense outdoor physical activities (Zumba) at a fundraising event. Our patient had typical substernal chest pain induced with exercise and was relieved by sublingual nitroglycerin in the Emergency Department. The pain started after 2 h of intensive Zumba workout. On review of her history, our patient was noted to be taking spironolactone 125 mg once daily for hirsutism for the past year. Our patient denied any family history of cardiac disease or heart failure. She admitted to being a former occasional smoker and to drinking alcohol socially. She denied any illicit drug use. She works as a social worker, and reported that she does not experience much stress in her life and denied any “one big life-changing event” or any major stressful news. While in the Emergency Department, our patient was hemodynamically stable and an electrocardiography was performed and showed sinus rhythm with no ST elevation/depression but noted T-wave inversion in leads I and aVL, and T wave flattening in leads V1 and V2. Her troponin levels were 0.294 and 0.231 consecutively. An echocardiogram was done and showed hypokinetic apical and mid-distal walls and hyperdynamic basal walls of the left ventricle with an ejection fraction of 35–40%, consistent with apical ballooning syndrome. Cardiac catheterization was subsequently done and showed depressed left ventricle systolic function, ejection fraction of 30–35% with anteroapical dyskinesia and no evidence of coronary artery disease.

Our patient was diagnosed with Takotsubo cardiomyopathy after fulfilling all four of the Mayo Clinic’s diagnostic criteria and was subsequently treated with a beta blocker, and angiotensin-converting enzyme inhibitor.

**Conclusions:**

Our patient did not have one clear trigger for her overt Takotsubo cardiomyopathy other than the Zumba activity. Zumba is considered an activity with excessive sympathetic stimulation leading to catecholamine-induced microvascular spasm or through to direct myocardial toxicity, which is postulated to be behind the pathophysiology of Takotsubo cardiomyopathy. Another interesting finding in our patient was her use of spironolactone, as treatment for hirsutism, which is an aldosterone antagonist. Aldosterone actually potentiates the effects of catecholamine and thus activates the sympathetic system. Spironolactone can thus be considered as cardioprotective against the effects of catecholamine on the heart and that is why it is considered to be beneficial and subsequently improves mortality in chronic heart failure as described in several studies.

## Background

Takotsubo cardiomyopathy (TCM) or stress cardiomyopathy is characterized by transient left ventricular apical ballooning in the absence of coronary occlusion. Takotsubo cardiomyopathy was first reported in 1990 in Japan and was given the name “Takotsubo” after the Japanese octopus trap, due to the resemblance of its shape to the appearance of the patient’s left ventricle in systole. The underlying pathophysiological mechanism is still unclear but possible causes have been proposed mainly catecholamine cardiotoxicity, followed by metabolic disturbance, coronary microvascular impairment, and multivessel epicardial coronary artery vasospasm. Several diagnostic criteria have been proposed with the most commonly used one being the Mayo Clinic 2004 diagnostic criteria that was modified in 2008: (1) transient hypokinesis, akinesis, or dyskinesis of the left ventricular mid segments with or without apical involvement; (2) absence of obstructive coronary disease or angiographic evidence of acute plaque rupture; (3) new electrocardiographic abnormalities or modest elevation in cardiac troponin; (4) absence of: pheochromocytoma and myocarditis. Takotsubo cardiomyopathy accounts for 1–2% of patients presenting with acute coronary syndrome with the majority of patients diagnosed with Takotsubo cardiomyopathy being women > 55 years of age [1, 2].

Here, we discuss the case of a 38-year-old woman presenting with typical chest pain, electrocardiography (EKG) changes and cardiac markers consistent with acute coronary syndrome (ACS), who was subsequently diagnosed with TCM without having a clear trigger.

## Case presentation

On August 1, 2017, a 38-year-old American woman with a negative past medical history presented to our Emergency Department (ED) with chest pain. Our patient stated that pain started on the morning of July 31 around 11:00 am, while participating in outdoor physical activities (Zumba) at a fundraising event. After 2 h into the workout, she began to feel chest pain, that was substernal, pressure-like, and throbbing in nature. The pain was non-radiating, six out of ten in intensity, and associated with diaphoresis and shortness of breath. Pain was alleviated in the ED when she was given sublingual nitroglycerin and intravenous morphine. She also stated episodes of a “fluttery feeling” and at times feeling lightheaded. She denied any previous history of a similar episode. She has no known cardiac history. Our patient did state that she never did any kind of exercise except for the activities of daily living. On review of her medical history, she stated that she was taking spironolactone for hirsutism, from August to January 2016 and then May 2017–present, with initial dosing of 125 mg titrated up to 150 mg and then back down to 125 mg due to muscle fatigue. Our patient had intentional weight loss of 30 lbs from March to July 2017. She follows a healthy diet and has a normal body mass index (BMI) of 24. Our patient denied any family history of cardiac disease or heart failure. She admitted to being a former occasional smoker and to drinking alcohol socially. She denied any illicit drug use. She works as a social worker, and reported that she does not experience much stress in her life and denied any “one big life-changing event” or any major stressful news. She reported a history of anxiety and was medicated approximately 4 years ago with alprazolam that was later discontinued as our patient was comfortable with her stress levels at that time.

While in the ED, patient was hemodynamically stable with a heart rate of 60s–70s beats/min, respiratory rate of 12–20 breaths/min, blood pressure of 90s/60s mmHg, O_2_ saturation of > 95% on room air, and afebrile. On physical examination, our patient was awake, alert, oriented to self, others, time, and place. Her skin was warm, dry, with no apparent rashes. Her neck was supple and non-tender with no jugular venous distension or apparent masses. A cardiovascular examination showed a regular rate and rhythm, with no murmurs or gallops. Our patient did not demonstrate any lower extremity edema and pulses were intact bilaterally. Her lungs were clear with equal breath sounds. Her abdomen was soft and non-tender with no hepatosplenomegaly. No lymphadenopathy was appreciated. A neurological examination showed grossly intact cranial nerves 2–12, normal sensation, strength was full bilaterally, normal reflexes, intact coordination and normal gait. An electrocardiogram (EKG) was performed and showed sinus rhythm with no ST elevation/depression but noted T-wave inversion in leads I and aVL, and T wave flattening in leads V1 and V2. Her troponin levels were 0.294 and 0.231 consecutively. A chest X-ray showed mild atelectasis at the left lung base, prominent pectus excavatum deformity of the anterior chest wall, bilateral small pleural effusions or pleural scarring, and no evidence of pulmonary vascular congestion.

**Fig. 1:**
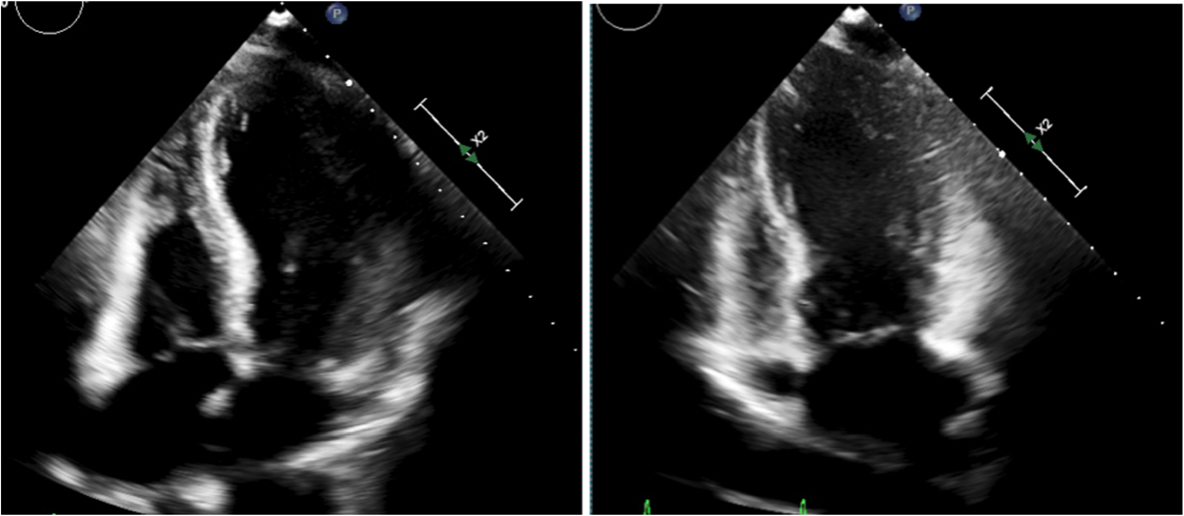
Echocardiogram of the patient showing hypokinetic apical and mid-distal walls and hyperdynamic basal walls of the left ventricle

An echocardiogram was done and showed hypokinetic apical and mid-distal walls and hyperdynamic basal walls of the left ventricle (LV) with ejection fraction (EF) of 35–40%, consistent with apical ballooning syndrome (Fig. 1).

**Fig. 2:**
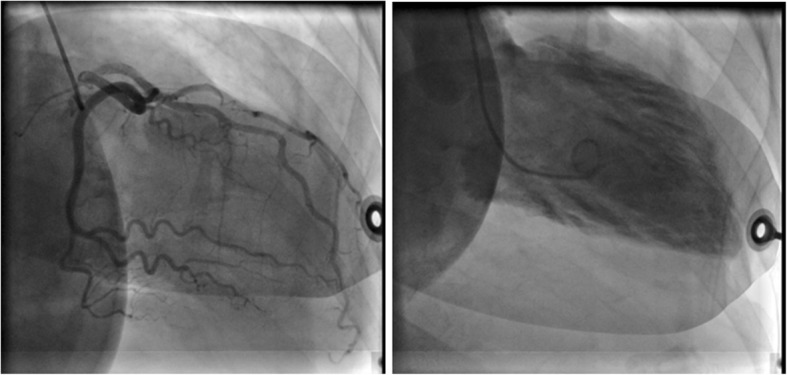
Cardiac catheterization of the patient showing patent coronary vessels with anteroapical dyskinesia

Cardiac catheterization was subsequently done and showed depressed LV systolic function, ejection fraction of 30–35% with anteroapical dyskinesia and no evidence of coronary artery disease (CAD) (Fig. 2).

Our patient was subsequently treated with a beta blocker, and angiotensin-converting enzyme inhibitor. Our patient was discharged with a LifeVest (a wearable cardiac defibrillator) and instructed to follow up in 1 month with the cardiologist to have a repeat echocardiogram. One month later, during her follow-up with the cardiologist, she denied any episodes of chest pain, palpitations, or shortness of breath. At that visit, a repeat echocardiogram was done and showed normal findings with an EF of 60–65%, thus the LifeVest was discontinued. After that visit, patient was lost to follow-up. We followed CARE reporting guidelines in publishing our case report with important information from our case presented as a timeline (Table 1).

**Table 1:** Timeline table

Relevant past medical history and interventions		
Negative past medical history. She was taking spironolactone for hirsutism, from August–January 2016 and then May 2017–present, with initial dosing of 125 mg titrated up to 150 mg and then back down to 125 mg due to muscle fatigue.		
Summaries from initial and follow-up visits	Diagnostic testing	Interventions
After 2 h into the workout, she began to feel chest pain that was substernal, pressure-like, and throbbing in nature. The pain was non- radiating, six out of ten in intensity, and associated with diaphoresis and shortness of breath. Pain was alleviated in ED when she was given sublingual nitroglycerin and intravenous morphine.	EKG, chest X-ray, echocardiogram.	Cardiac catheterization.
Treated with a beta blocker, and angiotensin-converting enzyme inhibitor. Patient was discharged with a LifeVest (a wearable cardiac defibrillator).		
Follow-up visit in 1 -month with a repeat echocardiogram showed normal findings with an EF of 60–65%, thus the LifeVest was discontinued. After that visit, patient was lost to follow-up.		

*EF* ejection fraction, *EKG* electrocardiography

## Discussion

Our patient was diagnosed with Takotsubo cardiomyopathy after fulfilling all four of the Mayo Clinic’s diagnostic criteria [1]. Takotsubo cardiomyopathy is also known as stress-induced cardiomyopathy (SIC), since the main provoking factor is thought to be the stress-induced release of catecholamines, specifically norepinephrine (NE) [3]. Our patient did not have one clear trigger for her overt TCM other than the Zumba activity. Our patient was doing Zumba, an excessive exercise activity, when she developed the symptoms. Zumba is considered an activity with excessive sympathetic stimulation, which is postulated to be behind the pathophysiology of Takotsubo cardiomyopathy through the effect of these catecholamine hormones that are released during exercise leading to catecholamine-induced microvascular spasm or through direct myocardial toxicity [1, 2]. On the other hand, another interesting finding in our patient was her use of spironolactone, as treatment for hirsutism, which is an aldosterone antagonist. Aldosterone actually potentiates the effects of catecholamine and thus activates the sympathetic system. Spironolactone can thus be considered as cardioprotective against the effects of catecholamine on the heart and that is why it is considered to be beneficial and subsequently improves mortality in chronic heart failure as described in several studies [4]. This could prompt further studies to better understand the pathophysiology behind the association between catecholamine and Takotsubo cardiomyopathy.

## Conclusions

Takotsubo cardiomyopathy is another cause of sudden cardiac death in young people especially among those with physical or mental stressors that exacerbate this condition. Takotsubo cardiomyopathy is still a complex entity with a complex and not well understood pathophysiology. Even though most of the young patients with Takotsubo cardiomyopathy recover or improve within 4–5 weeks, some patients do develop complications including: pulmonary edema, malignant arrhythmia, and even death. Therefore, Takotsubo cardiomyopathy is getting more and more attention and physicians need to keep a high suspicion for Takotsubo cardiomyopathy in young patients presenting with clinical manifestations similar to other cardiac diseases.

## Abbreviations

ACS: Acute coronary syndrome
BMI: Body mass index
CAD: Coronary artery disease
ED: Emergency department
EF: Ejection fraction
EKG: Electrocardiography
LV: Left ventricle
NE: Norepinephrine
SIC: Stress-induced cardiomyopathy
TCM: Takotsubo cardiomyopathy

## References

[1] Ono R, Falcão L. Takotsubo cardiomyopathy systematic review: pathophysiologic process, clinical presentation and diagnostic approach to Takotsubo cardiomyopathy. Int J Cardiol. 2016;209:196–205. 10.1016/j.ijcard.2016.02.012. 26896623

[2] Wang Y, Xia L, Shen X, *et al*. A new insight into sudden cardiac death in young people: a systematic review of cases of Takotsubo cardiomyopathy. John S, ed. Medicine. 2015;94(32):e1174. 10.1097/MD.0000000000001174. PMC4616674 26266349

[3] Duric V, Clayton S, Leong M, Yuan L. Comorbidity factors and brain mechanisms linking chronic stress and systemic illness. Neural Plast. 2016;2016:1–16. 10.1155/2016/5460732. PMC4761674 26977323

[4] Struthers A. Why does spironolactone improve mortality over and above an ACE inhibitor in chronic heart failure? Br J Clin Pharmacol. 2001;47(5):479–82. 10.1046/j.1365-2125.1999.00954.x. PMC2014181 10336570

